# Clinical and epidemiological analysis of basosquamous carcinoma: results of the multicenter study

**DOI:** 10.1038/s41598-020-72732-x

**Published:** 2020-10-28

**Authors:** Magdalena Ciążyńska, Martyna Sławińska, Grażyna Kamińska-Winciorek, Dariusz Lange, Bogumił Lewandowski, Adam Reich, Marta Pabianek, Katarzyna Szczepaniak, Adam Hankiewicz, Małgorzata Ułańska, Jan Morawiec, Maria Błasińska-Morawiec, Zbigniew Morawiec, Janusz Piekarski, Robert Brodowski, Anna Zaryczańska, Michał Sobjanek, Witold Owczarek, Monika Słowińska, Katarzyna Wróbel, Andrzej Bieniek, Anna Woźniacka, Małgorzata Skibińska, Joanna Narbutt, Wojciech Niemczyk, Karol Ciążyński, Aleksandra Lesiak

**Affiliations:** 1 Department of Proliferative Diseases, Nicolaus Copernicus Multidisciplinary Centre for Oncology and Traumatology, ul. Pabianicka 62, 93-513 Łódź, Poland; 2 Department of Dermatology, Venereology and Allergology, Medical University of Gdańsk, Gdańsk, Poland; 3 Department of Bone Marrow Transplantation and Hematology-Oncology, The Maria Skłodowska-Curie Memorial Cancer Centre and Institute of Oncology, Branch in Gliwice, Gliwice, Poland; 4 University of Technology, Faculty of Medicine, Rolna 43, 40-555 Katowice, Poland; 5 Clinical Department of Maxillo-Facial Surgery, Frederic Chopin Provincial Specialist Hospital, Rzeszów, Poland; 6 Department of Dermatology, University of Rzeszów, Rzeszów, Poland; 7 Department of Surgical Oncology, Nicolaus Copernicus Multidisciplinary Centre for Oncology and Traumatology, Łódź, Poland; 8 Department of Surgical Oncology, Chair of Oncology, Nicolaus Copernicus Multidisciplinary Centre for Oncology and Traumatology, Medical University of Łódź, Łódź, Poland; 9 Dermatology Clinic, Military Institute of Medicine in Warsaw, Warsaw, Poland; 10 Centrum Medyczne Bieniek, Wrocław, Poland; 11 Department of Dermatology and Venereology, Medical University of Łódź, Łódź, Poland; 12 Department of Dermatology, Paediatric Dermatology and Oncology Clinic, Medical University of Łódź, Łódź, Poland; 13 Department of Analysis and Strategy, National Health Fund, Warsaw, Poland; 14 Institute of Applied Computer Science, Lodz University of Technology, Łódź, Poland

**Keywords:** Cancer epidemiology, Head and neck cancer, Skin cancer

## Abstract

Basosquamous carcinoma (BSC) is a rare non-melanoma skin cancer that shares the characteristic features of both basal and squamous cell carcinomas (BCC, SCC). Our research enables better characterization of BSC in comparison to high-risk subtypes of BCC and SCC. Paper includes a retrospective analysis of BSC cases regarding sex, age, number of tumors and anatomical distribution in comparison to BCC and SCC evaluating the differences and defining the implications. Histologically confirmed carcinomas recorded between 1999 and 2019 were studied. 181 diagnosed BSC cases were identified, making this study the largest cohorts of BSC patients reported worldwide. Most cases were reported on head and neck. Analysis of facial anatomic distribution shows that most commonly affected sites were the nose (43%) and the cheek (25%). The age at excision of metatypical BCC was higher than those of low-risk BCC (*P* < 0.05), however similar to high-risk BCC (*P* = 0.20). We revisited that the concept of BSC is the most similar to high-risk subtypes of BCC. Patients with diagnosed BSC have higher risk of second nonmelanoma skin cancer. Therefore, the frequency of follow-up examination should be adjusted to the individual risk of another skin cancer.

## Introduction

Basal cell carcinoma (BCC) followed by squamous cell carcinoma (SCC) are the two most common subtypes of nonmelanoma skin cancer (NMSC) affecting white-skinned individuals. Recently a significant world-wide increase in incidence of both cancers has been observed^1–4^. Although BCC and SCC share many similarities, they have important etiological differences which cause the substantial variation between these skin cancers’ incidence rates. Generally, NMSC is curable and BCC has minimal potential risk of metastasis, with an estimated incidence between 0.00281% and 0.5%^5^. Mortality rate for BCC is exceptionally low, while SCC has a higher metastatic rate estimated on 0.5–16% and higher related mortality^6^. Moreover, recent studies suggest that BCC is not a homogeneous entity and that specific histological subtypes show different clinical behavior, occur at certain body areas and might have different etiology and recurrence potential after standard excision^7^. Superficial BCCs are predominantly located on the trunk, whereas more common nodular type is mainly observed on the head and neck^7^, most likely due to more frequent intermittent UV exposure. This is confirmed by the fact that both intermittent and intense sun exposure are the leading risk factors of BCC^8^.

Morpheaform BCC is less common than superficial and nodular variants^9^ and this subtype is known to be more aggressive than other types, because of its tendency to infiltrate deep subcutis. Similarly, infiltrative BCC is considered a high-risk histopathological subtype because it is more likely to be incompletely excised or occurs especially on face where surgical margins might be conservative^10–12^.

**Figure 1:**
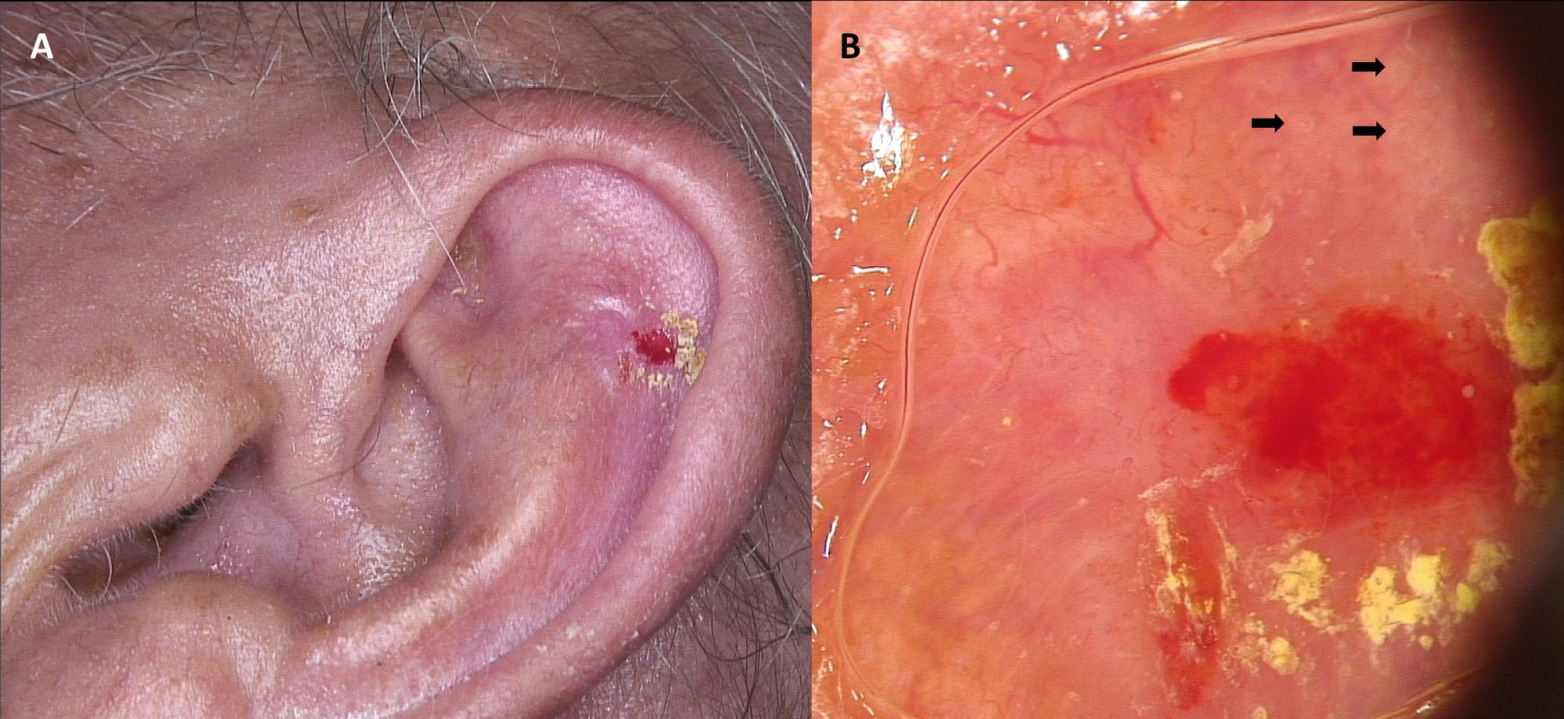
Ulcerated basosquamous carcinoma of the left auricule (**A**). Dermoscopy shows central red and yellow structureless areas corresponding with the presence of ulceration and crust, respectively. On the periphery white circles (black arrows), arborizing vessels ad well as polymorphic vessels on the white-pinkish background may be observed (**B**).

**Figure 2:**
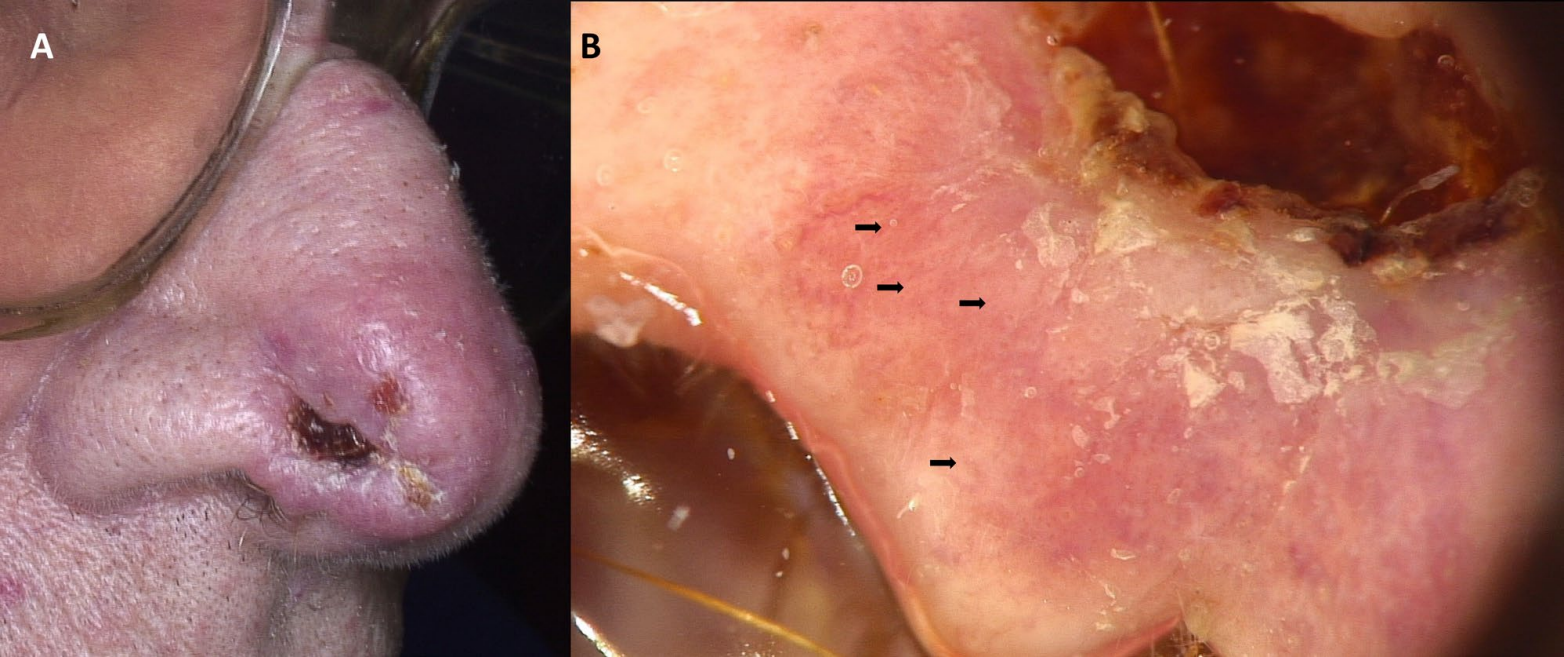
Ulcerated basosquamous carcinoma of the nose (**A**). Dermoscopy shows central crust covering an ulceration. On the periphery white circles (black arrows), white scale, as well as unfocused serpentine vessels may be observed (**B**).

**Figure 3:**
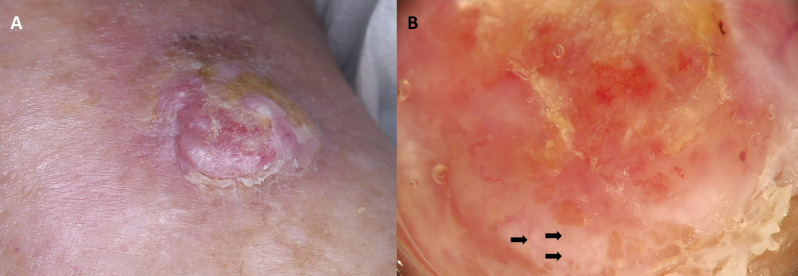
Basosquamous carcinoma of the left lower leg (**A**). Dermoscopy shows white circles (black arrows), white-pinkish structureless areas, white-scale, whitish-yellowish crust as well as polymorphic vascular pattern (**B**).

Metatypical basal cell carcinoma synonymously labelled basosquamous carcinoma (BSC) is a rare cutaneous neoplasm that has caused considerable controversy concerning its classification and pathogenesis^13^. The diagnosis of BSC is confirmed by histopathological examination. Clinical differentiation from other BCC subtypes is difficult, although dermoscopic evaluation may provide some important clues^14^. The dermoscopic pattern of BSC combines characteristics of both BCC and SCC including unfocused arborizing vessels, white structureless areas, keratin masses, ulceration or blood crusts, white structures, blue-gray blotches and blood spots on keratin masses (Figs. 1, 2, 3). Detection of at least one overlapping dermoscopic criterion both of BCC and SCC is an alarming sign for BSC in dermoscopy^15^. This is crucial, especially because the non-specific clinical presentation of BSC may extend time to diagnosis and lead to underestimation of possible later consequences such as local recurrence and potential lymph node or distant metastasis. Basosquamous carcinoma is more aggressive and invasive than both BCC and probably also SCC, although the latter assumption is controversial^16–20^. Despite benign appearance, BSC is characterized by aggressive subclinical spread with higher rates of recurrence: 12–51% for surgical excision and 4% for Mohs micrographic surgery. Moreover, BSC has a relatively high metastatic potential, estimated on 5–10%^21,22^.

Although from the first case description of a carcinoma with abutting basaloid and squamous features, without clear separation in 1894 is over hundred years, it remains unclear whether BSC should be classified as a BCC or SCC^23,24^. Initially, it was suggested that these lesions represent a collision of separate primary BCC and SCC which closely develop opposed to each other^23,25^. Summarization theory, which suggests that BSC is basal cell carcinoma that undergoes squamous differentiation^21^ is supported by the current broad definition provided by the World Health Organization (WHO) in Classification of Tumors, that have categorized BSCs as “basal cell carcinomas that are associated with squamous differentiation”. On the other hand, National Comprehensive Cancer Network (NCCN), states that some cases of basosquamous tumors may *be prognostically similar to SCC, so that clinicopathologic correlation is recommended in these cases* and these patients may require different approach with respect to treatment and follow-up compared to BCC patients. Due to an aggressive biological behavior and clinical course that distinguishes BSC from other forms of BCC, the use of term “metatypical basal cell carcinoma” may be misleading and is not recommended by some authors^22^.

This study was undertaken to learn more about characteristic epidemiological features of basosquamous carcinoma in patients treated surgically in a defined period. Moreover, we compared BSC with the general characteristics of BCC and SCC.

## Methods

All cases included in the study were histologically confirmed BCCs, SCCs or BSCs consecutively admitted and surgically excised at the seven sites in Poland: Gdansk, Gliwice, Lodz (2 sites), Rzeszow, Warsaw and Wroclaw, during a 21-year period (1999–2019).

All patients were Caucasians with skin phototype between I and III, diagnosed and treated either in a public hospital or in a private practice. Information recorded was the patient’s age, gender, anatomical localization of the cancer and histological diagnosis. Excision margins, the presence of lymphovascular or perineural invasion, AJCC pT stage, and the status of reexcision specimens were also documented when data was available.

Study approved by Human Research Ethics Committee of the Medical University of Lodz, Poland (RNN/209/18/KE) and informed consent was obtained from all organizations the subjects’ data is being sourced from. Written consent was not obtained from the study participants because the retrospective data used encrypted identification of the individuals. All methods and procedures were conducted in accordance with relevant guidelines and regulations as well as with the updated Declaration of Helsinki. This study was given a formal waiver for the need for consent by the Institutional Review Board of Medical University of Lodz.

The tumor excision was performed with standard excision or histographic surgery (Mohs micrographic surgery) in all operated cases. The tumors described by histopathologists as basosquamous carcinoma (BSC), basosquamous cell carcinoma, metatypical basal cell carcinoma, basal cell carcinoma with squamous differentiation, metatypical basal cell carcinoma as basosquamous have been analyzed. All histological specimens were excised and examined primarily by a dermatopathologist using haematoxylin and eosin (H + E) staining as a minimum. A small proportion of specimens were subsequently subjected to immunohistochemistry analysis using an anti-human epithelial antigen monoclonal antibody BerEP4, where the diagnosis was less clear. We used the following strict histologic criteria for the diagnosis of BSC: loss of BerEP4 staining in portions of the tumor, loss of palisading, increased nuclear atypia, significant squamous differentiation, and loss of basaloid cytology in portions of the tumor.

Recurrent tumors were excluded from the study. In tumors where multiple subtypes are noted, all subtypes are listed in the final diagnosis. Patients who had two or more tumors diagnosed were counted as two or more tumor cases. The data were compared with the same results obtained for BCCs and SCCs. The subtype classification was according to the National Comprehensive Cancer Network (NCCN) guidelines v1.2020 (low-risk BCC histologic subtypes include superficial, nodular, keratotic, infundibulocystic, and fibroepithelial BCC; high-risk BCC subtypes include infiltrative, sclerosing/morpheaform, micronodular, and BCC with carcinosarcomatous differentiation; high-risk SCC histologic subtypes include acantholytic, adenosquamous, desmoplastic, or carcinosarcomatous; low-risk SCC histologic subtypes include verrucous and keratoacanthomatous SCC).

All results were analyzed statistically with Statistica 13.0 (Statsoft, Kraków, Poland). The obtained results were analyzed statistically by the Student's t-test, analysis of variance (ANOVA) post hoc comparisons and χ^2^ test with Yates correction if needed. *P*-values less than 0.05 were considered as statistically significant.

## Results

181 cases (women:men ratio: 0.83:1) reported in 168 patients (76 women and 92 men) with basosquamous carcinoma tumor were included in the study. In the selected cohort of patient in the analyzed period, SCC was found in 2065 cases (1020 men and 1045 women) including high-risk SCC in 162 cases (72 men and 90 women) and low-risk SCC in 814 cases (402 men 412 woman) and BCC in 11,848 cases, including high-risk BCC in 769 cases (373 men and 396 women) and low-risk BCC in 4032 cases (1958 men 2074 woman). BSC composed 2.1% of all NMSC.

The mean age of patients differed for various NMSC types. Patients with low-risk BCC were younger (68.7 ± 12.8 years) than patients with BSC (72.2 ± 11.5 years) and this difference was statistically significant (*P* < 0.05), while patients with SCC were significantly older than patients with BSC (*P* < 0.05). However, the average age of patients with high-risk BCC (71.1 ± 12.3 years) was similar to patients with BSC with no statistical significance (*P* = 0.20).

Women to men predominance was observed for low-risk SCC cases (1.02:1), high-risk SCC cases (1.25:1), low-risk BCC cases (1.06:1) and high-risk BCC cases (1.06:1), while opposite observation was noted for BSC cases (0.83:1) with the significant predominance of men (*P* < 0.05).

In most cases lesions were pT1 [≤ 20 mm] (n = 102), followed by pT2 [> 20 mm to ≤ 40 mm] (n = 78) and pT3 [> 40 mm] (n = 3), with 3 lesions displaying lymphovascular invasion and 5 lesions with perineural invasion. Mean excision margins were 3.8 mm peripherally (SD ± 2.1 mm). The inadequate excision rate (defined as resection margins < 1 mm or involved margins) was observed for 43 cases (24%), mostly on facial area.

Single, excised and histologically confirmed BSC presented 100 patients. Multiple carcinomas were diagnosed in 68 patients with BSC (40%) and it is significantly higher rate in comparison to SCC (26%, *P* < 0.05) and BCC (23%, *P* < 0.05). Usually, BSCs were diagnosed 3 months after the diagnosis of high-risk BCC, while BSC occurred prior (3 months) to the first diagnosed low-risk BCC. In this context, no correlation has been observed for SCC.

In both sexes, anatomical distribution of all types of tumors was dominated by the face, with increased rate of BSC (86% for men, 89% for women) in comparison to other types of NMSC (*P* < 0.05). The second most frequent location was the trunk for BSC (6% for men, 8% for women). Table 1 presents anatomical distribution of NMSC according to gender.

**Table 1:** Clinical characteristics of patients with NMSC.

	Low-risk SCC		High-risk SCC		BSC		Low-risk BCC		High-risk BCC	
Total number of lesions	814		162		181		4032		769	
mean age	77.3^a^		76.5^a^		72.2^a^		68.7^a^		71.1^a^	
Sex ration (F/M)	1.02		1.25		0.83^b^		1.06		1.06	
**Men**										
Face	225	(*61%*)	47	(*85%*)	81	(*86%*)	549	(*57%*)	269	(*76%*)
Trunk	46	(*13%*)	0	(*0%*)	6	(*6%*)	263	(*27%*)	31	(*9%*)
Upper limb	32	(*9%*)	2	(*4%*)	2	(*2%*)	62	(*6%*)	11	(*3%*)
Lower limb	41	(*11%*)	0	(*0%*)	0	(*0%*)	44	(*5%*)	4	(*1%*)
Scalp	11	(*3%*)	2	(*4%*)	2	(*2%*)	20	(*2%*)	25	(*7%*)
Neck	13	(*4%*)	4	(*7%*)	3	(*3%*)	51	(*5%*)	13	(*4%*)
n/d	34		17		5		969		20	
all	402		72		99		1958		373	
**Women**										
Face	275	(*67%*)	82	(*92%*)	68	(*89%*)	549	(*57%*)	296	(*79%*)
Trunk	27	(*7%*)	0	(*0%*)	6	(*8%*)	248	(*26%*)	37	(*10%*)
Upper limb	23	(*6%*)	1	(*1%*)	0	(*0%*)	48	(*5%*)	7	(*2%*)
Lower limb	57	(*14%*)	2	(*2%*)	0	(*0%*)	70	(*7%*)	7	(*2%*)
Scalp	2	(*0%*)	1	(*1%*)	1	(*1%*)	25	(*3%*)	17	(*5%*)
Neck	12	(*3%*)	3	(*3%*)	1	(*1%*)	55	(*6%*)	11	(*3%*)
n/d	4		1		6		1079		21	
All	412		90		82		2074		396	

Data are presented as n (%). n/d: not defined.

BCC, basal cell carcinoma; SCC, squamous cell carcinoma; BSC, basosquamous cell carcinoma; F/M, female to male ratio.

^a^The mean age of patients differs significantly between BSC and other subtypes (*P* < 0.05).

^b^Men to women predominance was statistically significant for BSC (*P* < 0.05).

In most cases (149) BSC was located on the face, especially on the nose (44% for men, 42% for women). Other locations on the face were as follows: the cheek (20% for men, 27% for women), the eyehole (15% for men, 21% for women), and the earlobe (16% for men, 6% for women). Body-site distribution of BSC was strongly dominated by the face in comparison to other types of NMSC (*P* < 0.05). Table 2 presents distribution of tumors on the face.

**Table 2:** NMSC subtypes localization on face based on sex.

	Low-risk SCC		High-risk SCC		BSC		Low-risk BCC		High-risk BCC	
**Men**										
Nose	28	(*21%*)	12	(32%)	24	(*44%*)	204	(*49%*)	24	(*44%*)
Eyehole	6	(*4%*)	2	(5%)	8	(*15%*)	17	(*4%*)	7	(*13%*)
Cheek	41	(*30%*)	9	(24%)	11	(*20%*)	153	(*37%*)	9	(*17%*)
Temple	1	(*1%*)	1	(3%)	0	(*0%*)	9	(*2%*)	1	(*2%*)
Forehead	1	(*1%*)	0	(0%)	0	(*0%*)	0	(*0%*)	0	(*0%*)
Earlobe	42	(*31%*)^a^	9	(24%)^a^	9	(*16%*)^a^	19	(*5%*^)^	9	(*17%*^)a^
Lips	15	(*11%*)	5	(13%)	2	(*4%*)	7	(*2%*)	2	(*4%*)
Chin	2	(*1%*)	0	(0%)	1	(*2%*)	8	(*2%*)	2	(*4%*)
n/d	89		9		26		133		215	
All	225		47		81		549		269	
**Women**										
Nose	46	(*28%*)	20	(*36%*)	20	(*42%*)	263	(*53%*)	46	(*60%*)
Eyehole	3	(*2%*)	2	(*4%*)	10	(*21%*)	29	(*6%*)	5	(*6%*)
Cheek	94	(*58%*)	25	(*45%*)	13	(*27%*)	163	(*33%*)	13	(*17%*)
Temple	2	(*1%*)	1	(*2%*)	0	(*0%*)	10	(*2%*)	3	(*4%*)
Forehead	1	(*1%*)	0	(*0%*)	0	(*0%*)	0	(*0%*)	0	(*0%*)
Earlobe	5	(*3%*)^a^	2	(*4%*)^a^	3	(*6%*)^a^	9	(*2%*)	4	(*5%*)^a^
Lips	10	(*6%*)	3	(*5%*)	2	(*4%*)	2	(*0%*)	0	(*0%*)
Chin	2	(*1%*)	2	(*4%*)	0	(*0%*)	20	(*4%*)	6	(*8%*)
n/d	112		27		20		53		219	
All	275		82		68		549		296	

Data are presented as n (%). n/d: not defined.

BCC, basal cell carcinoma; SCC, squamous cell carcinoma; BSC, basosquamous cell carcinoma.

^a^More lesions on earlobe occurring in men than women (*P* < 0.05).

## Discussion

Non-melanoma skin cancer represents the most common group of skin neoplasms in white population with an increasing incidence worldwide. Unfortunately, cases of BCC and SCC, unlike other cancers, are often not tracked or even excluded in cancer registries and statistical analyses. The most likely reasons for this are that NMSC are associated with lower mortality compared to other malignancies. However, they stand for social, cosmetic and economical issue, therefore, studying and understanding their current epidemiological trends is considered crucial in order to create a public health strategy to achieve early and adequate control of the disease.

BSC is a rare NMSC type, currently representing approximately 1.2% to 2.7% of all malignancies in this group^13,16,22,24,25^. However, it is also suspected to be underdiagnosed, partially due to non-representative biopsy specimens taken, as often characteristic histological features are present in deep layers only. In our study BSC was reported in 2.1% of all cases confirming the relative rarity of this tumor.

Recent studies showed that some variants of BCC and SCC are more aggressive than others^26,27^. It has been suggested that infiltrative and morpheaform BCC subtypes are generally regarded high-risk subtypes, either because of the higher risk of recurrence or due to their aggressive behavior^28^. In the opposite, superficial, nodular, and adenoid BCCs are considered less aggressive (low-risk) variants of BCC, with more indolent course. Well-differentiated histologic SCC subtypes with low metastatic potential include keratoacanthoma and verrucous carcinoma. In contrast, desmoplastic and adenosquamous SCCs are highly infiltrative, have a high risk of local recurrence, metastasis, and death^29^. Some authors believe that BSC is a variant of BCC, whereas others have suggested that BSC behaves more aggressively like SCC^21^.

In our study most cases of primary BSC excision of pT1 and pT2 tumors have good clinical outcome with none of the patients developing disease recurrence or metastasis. Positive margin is a significant risk factor for recurrence of a disease^16,21^. In most cases reported inadequate excision rate was associated with facial area. Tumors developed on face lead to challenges in resection and reconstruction, issues to achieve adequate surgical margin, and problems in providing a decent aesthetic outcome.

Another risk factor for recurrence of the disease is a perineural invasion^16,25^. However, only 5 patients in our study had histological evidence of perineural invasion, but no recurrence has been seen in the current study.

It is confirmed that UVR exposure is the most important risk factor of BCC, SCC and BSC^30^ and therefore, it is not surprising that the vast majority of these cancers occurs on the sun exposed areas such as the head compared to the other body sites^31^. Our research confirms that BSC and high-risk BCC and SCC tumors occur more frequently on the head and neck in comparison to low-risk BCC and SCC tumors, which are often located on the trunk and the limbs. However, the occurrence of low-risk BCC and SCC on the trunk was higher than aggressive subtypes of those tumors. There was a similarity in the anatomical localization between BSC and more aggressive subtypes of both BCC and SCC.

The results of other studies show that great majority of BSC cases are seen on the head and neck, mainly involving the central face^16,24,25,32^. In the series reported by Leibovitch et al.^25^ most tumors (170 out of 178, 95.6%) were located on the head and neck, with 59 patients (33.1%) having it on the nose. Other authors revealed the head and neck location in 82%^16^, 96%^24^, or even 97%^32^. Anatomical distribution in our cohort was similar, with 92% of tumors being located on the head and neck region. The most common primary tumor site on face was the nose that is similar to results provided by Wermker et al.^33^ that analyzed BSC exclusively on head and neck region.

It is also well established that BSC occurs more frequently in men^21,25^. Our study reflects similar results—male patients constituted 57%. However, when considering the occurrence of BSC on the ear only, we demonstrated that the gender disparity increased (Fig. 1). In our study, 79% of the BSCs occurring on the ear were diagnosed in men (*P* < 0.05). This finding is most likely attributable to the fact that it is more common for women to have hair covering the ears, thus shielding this anatomic site from ultraviolet radiation and making it relatively protected compared to the men’s ear^30^. What is more, great disparity of tumor type has been observed in term of lesions located on the earlobe in men. However, the great predilection for earlobe showed low-risk SCC, constituting 31% of the all low-risk SCC facial lesions, followed by the aggressive SCC subtypes (22%), aggressive BCC subtypes (17%) and BSC (16%). In contrast, only 5% of facial low-risk BCC were located on the ear.

Although patients usually presented in the six to eight decade of life^17,21,32^, the age range may be much wider, and there are reports of tumors occurring in the second decade^21^. We reported all patients at presentation between 18 and 102 years, while patients’ age with BSC lesions ranged from 46 to 92. The mean age at diagnosis for all BSC cases was 72 ± 11.5 years, which is similar to other high-risk BCC lesions, with mean age of 70.1 ± 12.3 years (*P* > 0.05). We have noticed an increasing proportion of SCC in older age groups (*P* < 0.05), and low-risk BCC in younger age groups in comparison to BSC (*P* < 0.05). The mean age of BSC was slightly higher compared to other studies^17,32^.

It is known that 30% to 50% of patients diagnosed with primary NMSC within the following 5 years will develop another similar outbreak of NMSC, which indicates that these patients are 10 times more likely to develop second BCC or SCC, compared to the general population. They are also more likely to develop melanoma. In our series 40% of patients diagnosed with BSC had associated another skin neoplasm, what is significantly higher in comparison to overall reported NMSC cases, where 23% represent patients with multiple lesions (*P* < 0.05). We reported the correlation between both low- and high-risk BCC and presentation of BSC lesions in single patients. Typically, diagnosis of low-risk BCC is followed by BSC after in average 2.5 years. On the other hand, usually BSC in average occurs 2.5 years before the presentation of high-risk BCC. No correlation has been observed between BSC and SCC presentation in single patients. These results confirm that BSC patients are more often than the general population diagnosed with new primary skin cancers. Therefore, the frequency of monitoring should be based on the risk of possible occurrence of another skin cancer. As we have shown, the period of the first two or three years of observation is the most critical, which is why patients at this time should undergo regular dermatological and dermoscopic examinations.

There are no clear guidelines concerning BSC management. Similarly to BCC and SCC, the first-line treatment option is surgical excision, but surgical margins should be wider than those for low-risk BCC due to the infiltrative growth pattern of this tumor. The aggressive behavior of BSC caused by high attention being paid to the role radiotherapy and Mohs' micrographic surgery in this neoplasm.

## Conclusions

To the best of our knowledge, this is the largest analysis of BSC worldwide which presents for the first time the detailed anatomical distribution, patients’ sex ratio and age, based on 181 histopathologically confirmed BSC in comparison to high- and low-risk BCC and SCC. We revisited the concept of BSC that is the most similar to high-risk subtypes of BCC and concluded that BSC, like morpheaform and infiltrative BCC subtypes, occurs more frequently on the head and neck compared to other locations. The reporting of the histopathological subtype of the BCC, BSC and SCC together with host demographics and body-site information should enhance clinical information and ultimately improve treatment regimens.

In contrast to previous data, the present study has shown that BSCs seam less aggressive and less likely to recur or metastasize. However, BSC tends to develop subsequent primary skin cancer more often than other types of NMSC. These data underline the need for frequent follow up with whole body surface examination for all patients diagnosed with BSC.

## Data Availability

The datasets generated during and/or analyzed during the current study are available from the corresponding author on reasonable request.

## Abbreviations

BCC: Basal cell carcinoma
BSC: Basosquamous cell carcinoma
NMSC: Non-melanoma skin cancer
SCC: Squamous cell carcinoma
